# Development and Evaluation of the Reliability and Validity of Video-Based Assessment Checklists of Nursing Skills via Chest-Mounted Cameras for Home-Visiting Nurses

**DOI:** 10.1155/nrp/7893018

**Published:** 2025-10-23

**Authors:** Sotaro Shimada, Toshiaki Takahashi, Aya Kitamura, Masaru Matsumoto, Yuko Mugita, Hiromi Sanada, Gojiro Nakagami

**Affiliations:** ^1^Department of Gerontological Nursing/Wound Care Management, Graduate School of Medicine, The University of Tokyo, Bunkyo, Tokyo, Japan; ^2^College of Nursing, University of Illinois Chicago, Chicago, Illinois, USA; ^3^Department of Adult Nursing, Yokohama City University, Yokohama, Kanagawa, Japan; ^4^Bureau of System Infrastructure Development, Japan Institute for Health Security, Shinjuku, Tokyo, Japan; ^5^Ishikawa Prefectural Nursing University, Kahoku, Ishikawa, Japan; ^6^Department of Adult Nursing, Ishikawa Prefectural Nursing University, Kahoku, Ishikawa, Japan; ^7^Department of Well-Being Nursing, Graduate School of Nursing, Ishikawa Prefectural Nursing University, Kahoku, Ishikawa, Japan; ^8^Global Nursing Research Center, Graduate School of Medicine, The University of Tokyo, Bunkyo, Tokyo, Japan

## Abstract

**Introduction:**

Ensuring that home-visiting nurses (HVNs) possess adequate skills for providing appropriate care is crucial in the context of increasing service demand with advanced care needs. However, current quality assurance relies on mentors' subjective assessments during accompanying visits, which are burdensome and rater-dependent. This study aimed to develop and evaluate the inter- and intrarater reliability and concurrent validity of objective video-based assessment (VBA) checklists for HVNs' nursing skills, focusing on peripheral intravenous catheter placement, pressure injury care, defecation care, and skin tear care to reduce the burden of skill assessment at home and ensure valid skill assessments.

**Methods:**

The checklists were developed through a literature review, focus group interviews, and a group meeting using the nominal group technique to ensure content validity. The inter- and intrarater reliabilities and concurrent validity of the checklists were evaluated using videos of nurses providing care to simulated patients. Five nursing researchers with experience in mentoring nurses assessed the videos twice using checklists with a washout period. The inter- and intrarater reliabilities were analyzed using Gwet's AC1 statistics. Concurrent validity was evaluated with sensitivity and specificity of assessment using checklists against raters' subjective assessments, with expertise in nursing skills as the gold standard.

**Results:**

The checklists demonstrated substantial to almost perfect inter- and intrarater reliabilities (AC1: 0.63–1.00) for each item. The overall assessments also showed more than substantial inter- and intrarater reliabilities (AC1: 0.72–1.00). The sensitivity and specificity of the checklists were both above 0.90, indicating high concurrent validity.

**Conclusion:**

The checklists were reliable and valid for objectively assessing HVNs' skills. The VBA suggests feasible and cost-effective methods for quality assurance, potentially improving the quality of home care. Further studies with patients in diverse clinical settings are required to enhance the generalizability of the results.

## 1. Introduction

The aging population has drastically increased the need for home care [1]. In Japan, which has the highest proportion of older adults, the number of clients using home-visiting nursing services is growing tremendously, and other countries are expected to follow this trend [2]. Home-visiting nurses (HVNs) are required to have the skills to provide assessment and care in a relatively short time because of time availability and difficulty in monitoring. Thus, it is imperative to ensure that HVNs have sufficient skills to provide appropriate care on time, especially for nurses with limited clinical experience, such as newly graduated nurses, as they recognize monitoring and clinical treatment as their weaknesses [3].

The current quality assurance of nursing skills in Japanese HVNs is mainly conducted based on subjective assessments made by mentors who accompany the visits and meetings to confirm the nursing skills of HVNs [4, 5]. However, the accompanying visits incur labor costs and are time-consuming. Consequently, only 55.7% of Japanese HVN agencies conduct continuous reassessments of nursing skills after only a few times of accompanying visits [5]. Thus, HVNs have limited opportunities to receive assessments of their nursing skills.

Although several quality indicators (QIs) in home care have been developed [6–8], most QIs focus on the comprehensive quality of care with an outcome- or structure-based approach according to the Donabedian model [9]. These QIs can measure only overall quality and are not suitable for the quality assurance of specific nursing skills. Furthermore, previous competency measurements specializing in nursing skills were developed for self-assessment and not for objective assessments [10]. Since there is a discrepancy between head nurses' assessment and nurses' self-assessment of competencies [11, 12], objective assessments are required for valid quality assurance.

However, to the best of our knowledge, there is no indicator that objectively assesses the nursing skills of an HVN to date. While objective structured clinical examination (OSCE), objective structured long examination record, Mini-Clinical Evaluation Exercise, and direct observation of procedural skills are often utilized in medical and nursing education worldwide to objectively assess their clinical competence [13–17], its implementation requires substantial human resources and financial costs [14, 18] with in-person assessment Given that HVN agencies typically have smaller staff sizes than hospitals, the implementation of those methods is considered unrealistic. Consequently, in the field of home care, there is a need to develop an alternative method for assessing nursing skills that is objective, quantitative, cost-effective, and less resource-intensive.

In summary, conventional methods, such as a mentor's subjective evaluations and self-assessment, are rater-dependent and unreliable, while major objective methods are resource-intensive. Compared to these methods, video-based assessment (VBA) tools may offer feasible and objective quality assurance. VBA is widely used by surgeons because of its objectiveness, convenience, and time efficiency [19]. In addition to convenience, VBA results are associated with short-term patient outcomes [20]. Thus, VBA can potentially be used to assure the quality of HVNs' nursing skills and reduce the number of accompanying visits for quality assurance.

We hypothesized that videos of providing nursing care to patients could be used to assess nursing skills. Among the different forms of VBA—such as checklists [21], global rating scales [22], and automated video analyses [23, 24]—we employed checklists because they allow step-by-step evaluation and provide specific, actionable feedback, which is essential for novice HVNs [25]. Our selection of nursing skills included peripheral intravenous catheter (PIVC) placement, pressure injury care, defecation care, and skin tear care, considering the prevalence of these conditions in home care and among older adults [26–30], difficulty in acquiring nursing skills [31], and the impact of incidence on the quality of life [32, 33].

This study aimed to develop VBA checklists and assess their inter- and intrarater reliability and concurrent validity.

## 2. Materials and Methods

### 2.1. Study Setting

**Figure 1:**
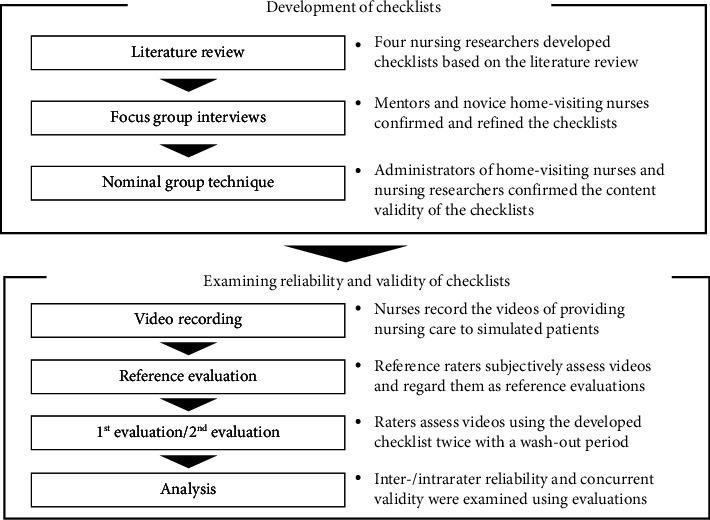
Procedure of development and evaluation of video-based assessment checklists of nursing skills.

This study consists of two phases: (1) a qualitative descriptive research design using a participatory approach for the development of the VBA tools and (2) an observational design for the evaluation of the inter- and intrarater reliability and concurrent validity of the developed VBA tools utilizing videos of nurses providing care to simulated patients (Figure 1). The study period was from April 2021 to December 2023.

### 2.2. Development of the Checklists

The concept of the tools in this study was to assess whether HVNs had sufficient skills to provide assessment and essential care about PIVC placement, pressure injury care, defecation care, and skin tear care to allow them to visit using video and nursing records. The checklists were expected to be used by mentors. The mentors assessed HVNs through the videos that were recorded while providing nursing care to patients and the nursing records of their assessments and care with the checklists.

The checklist development method was based on a literature review, focus group interviews (FGIs), and a group meeting using the nominal group technique (NGT). FGI is a frequently used method for collecting qualitative data from a specific population on a topic via semistructured interviews [34, 35]. NGT is a structured group-meeting technique for generating and prioritizing responses to specific questions by experts in the relevant field [36]. Our research group drafted the initial checklists through a literature review and included some elements. In initial drafts, each item was to be assessed as either “satisfactory” or “unsatisfactory,” and HVNs who received “satisfactory” on all items were to be considered “independent,” indicating they are competent enough to provide the nursing care during their visits. Subsequently, we conducted FGIs and NGT to refine and validate the contents of the checklists.

#### 2.2.1. FGIs

For the FGIs, we recruited mentors working in Japanese HVN agencies and novice HVNs working in Japanese HVN agencies with work experience as HVNs ranging from 1 to 4 years. The purpose of the FGIs was to refine and validate the initial draft of the checklist. The mentors were asked about: (1) whether the drafts of checklists fully covered the requirements that HVNs must fulfill in each nursing skill at their visits, (2) how many times accompanying visits are generally required for acquiring the nursing skills, and (3) the items in each nursing skill that are difficult for novice HVNs. Novice HVNs were asked about (1) whether the drafts of checklists fully covered the requirements that HVNs must fulfill in each nursing skill at their visits, (2) the items in each nursing skill that are difficult for novice HVNs, and (3) the cases and reasons the novice HVNs needed to ask for help from the mentor during the visits.

The FGIs were audio-recorded and transcribed verbatim. Three researchers (A.K., M.M., and T.T.) synthesized and coded the data using a qualitative descriptive approach [37]. After reaching consensus, the checklists were refined based on the FGIs, and participants subsequently reviewed the drafts to ensure the accuracy and validity of the codes.

#### 2.2.2. NGT

After the refinement of the draft through FGIs, a group meeting using the NGT was conducted. We recruited administrators working in Japanese HVN agencies and nursing researchers who were experts in home care nursing and gerontology nursing using convenience sampling. The purposes of the group meeting were as follows: (1) which of the items of each checklist were valid for inclusion in the skill assessment of HVNs and (2) whether the skill assessment using the videos and checklists was not too burdensome compared to the conventional assessment method. The participants in the FGIs did not participate in the meetings. H.S. conceived the group meetings, and G.N. played the role of a facilitator. The participants discussed extensively and approved at the meeting. The meeting continued until none of the participants made any further comments or corrections, and we identified the situation as a consensus. By obtaining consent from all the group-meeting participants, we ensured the content validity of the developed checklists.

#### 2.2.3. Translation of the Developed Checklists

The original version of the checklist was developed in Japanese, and Supporting 1 shows the English version. The translation was conducted by a professional English translator with expertise in the field, and another professional English translator checked for omissions or mistranslations. Finally, the authors carefully reviewed the translated checklists to confirm that no details or nuances were missing.

### 2.3. Evaluation of Reliability and Concurrent Validity of the Developed Checklists

The reliability and concurrent validity of the developed checklists were evaluated using videos, in which nurses provided care to simulated patients.

#### 2.3.1. Participants

We recruited nursing researchers as raters using convenience sampling for the evaluation. The researchers of this study also participated in the evaluations. The inclusion criteria were those registered nurses (RNs) who completed the Doctor of Nursing coursework and had experience in training or educating nurses. The exclusion criterion was the inability to communicate in Japanese. For each nursing skill, one of the raters, an expert in the nursing skill, was asked to play the role of a reference rater.

#### 2.3.2. Development of Videos

To record videos for the evaluation of the developed checklists, we recruited RNs with convenience sampling. The inclusion criteria were those aged ≥ 20 years with less than a year of experience as HVNs. The exclusion criterion was the inability to communicate in Japanese. As a result, nine RNs, including the researcher of this study, consented to participate in the research and were enrolled to obtain videos for assessments. Some RNs recorded more than one nursing skill.

**Figure 2:**
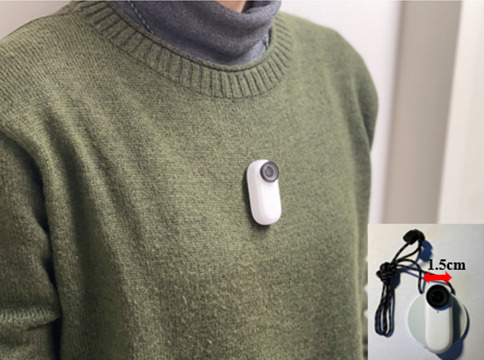
The chest-mounted camera used in the study. The camera (Insta360 GO2; Arashi Vision Inc., China) used in the study.

The videos were recorded using a small chest-mounted camera (Insta360 GO2; Arashi Vision Inc., China) (Figure 2). First, the RNs were briefly instructed on how to operate the camera. Before recording, the RNs were also given general information about the simulated patients, such as the purpose of using HVN services, comorbid diseases, current care plans, and activities of daily living with supplies for each nursing skill. Then, RNs were asked to provide the best possible care for the simulated patients following the provided information. It was also noted that in case of a lack of information, the RNs were supposed to ask the simulated patients in order to provide appropriate care.

**Figure 3:**
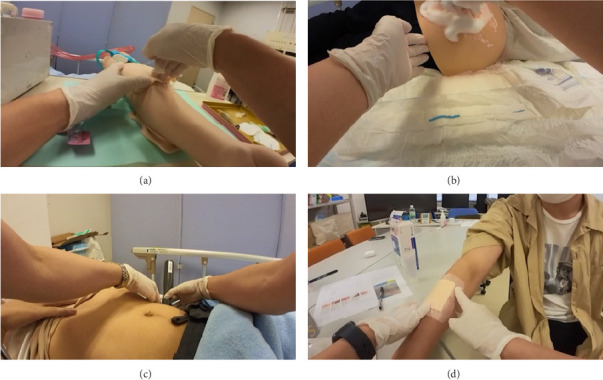
The scenarios of simulated patients. The scenarios of the simulated patients were explained prior to recording. The nurses were notified of the care to be provided and of basic information about the patients. However, there was a lack of information to provide appropriate care, and nurses were required to assess the condition of patients. The scenarios were as follows: (a) the scenario of peripheral intravenous catheter (PIVC) placement was that the patient had stage four breast cancer and was in the terminal stage after chemotherapy and axillary lymph node dissection on the left side of her body. Her swallowing function deteriorated, leading to aspiration pneumonia, which was treated with antibiotics. The nurse visits to place a PIVC for the antibiotics; (b) the scenario of pressure injury care was that the patient was bedridden and developed a pressure injury on the sacrum, in which the depth was D3, according to DESIGN-R 2020, after cerebral infarction with several prognostic symptoms. At the time of the visit, the nurse found that the pressure injury was deteriorating; (c) the defecation care scenario was that the patient had progressed to spinocerebellar degeneration, resulting in a decline in activities of daily living. At the time of the visit, the patient claimed that abdominal bloating caused constipation and asked the nurse for help. (d) The scenario of skin tear care was that the patient had rheumatoid arthritis and used home care for daily physical condition checks, pain management, and medication management. At the time of the visit, the patient had developed a skin tear.

We created simulated patient settings and scenarios and reached a consensus among the researchers. Simulated patients were played by S.S. and a graduate nursing student who was lectured on how to act by S.S. The scenarios for the simulated patients are briefly described in Figure 3.

Finally, seven videos were obtained for pressure injury care and skin tear care, and six videos were obtained for defecation care and PIVC placement. Hence, 26 videos in total were eligible to be assessed in this study. The videos obtained from the nurses were trimmed by the researcher to eliminate redundant scenes and minimize the burden on the raters.

#### 2.3.3. Procedure

The videos were shared using either cloud services or flash drives. The raters, including the reference rater, watched videos in randomized order to avoid bias and assessed the videos using the developed checklists. Only the reference rater of each nursing skill was asked to subjectively assess whether the nurses in the videos were sufficiently skilled to provide assessment and care during their visits. The assessments using the developed checklists were performed twice. We considered at least 1 week from the raters' first assessment as a washout period.

In cases of omission and misunderstanding of assessing the items or low reliability, the researcher confirmed the results of the assessment and assessment criteria for each rater and then permitted raters to change the assessment if mistakes were found.

The items that possessed low reliability, even after confirmation of the assessments, were revised based on the raters' opinions. The items were revised to clarify their criteria and make them affordable for assessment through the videos. In these cases, we were careful not to change the meaning of the items, and after the revisions were made, we confirmed the revised items with the NGT participants. After the revision, the raters were again asked to assess the revised items twice in total, with a washout period of 1 week. These revisions and reassessments were conducted continuously until all items achieved adequate reliability.

#### 2.3.4. Data Analysis

The participants' characteristics are presented as descriptive statistics. Categorical and continuous data are presented as numbers with frequencies and medians with interquartile ranges. A qualitative descriptive approach was conducted following a systematic process: transcription and organization of the data, generation of initial codes, annotation with analytic comments, identification of recurring patterns, development of thematic groupings, and linkage of the generalized findings to the established body of knowledge [37]. The inter- and intrarater reliabilities were calculated using Gwet's AC1 statistics [38, 39], which was chosen to avoid the paradoxical outcomes observed with kappa statistics. The inter-rater reliability was calculated using the first assessment through checklists, and intrarater reliability was calculated using each rater's first and second assessments. To interpret the reliability, Landis and Koch's benchmark was applied [40]. According to the interpretation, coefficients lower than 0 indicated poor agreement, 0–0.20 indicated slight agreement,  0.21–0.40 indicated fair agreement, 0.41–0.60 indicated moderate agreement, 0.61–0.80 indicated substantial agreement, and above 0.80 indicated almost perfect agreement. We regarded more than a substantial agreement as indicating adequate reliability for each item.

The concurrent validity was measured by calculating the sensitivity and specificity of the assessments using the developed checklists referenced with subjective assessments made by the reference rater, because no objective scale was found to measure the proficiency of nursing skills in home care. Sensitivity was calculated as the number of cases in which both the gold standard and the developed checklists assessed the performance as “independent,” divided by the total number of “independent” assessments according to the gold standard (i.e., subjective assessments made by the reference rater). Similarly, specificity was calculated as the number of cases in which both the gold standard and the developed checklists assessed the performance as “not independent,” divided by the total number of “not independent” assessments according to the gold standard. Following the rule of thumb for useful diagnostic tests, our goal was to achieve a combined sensitivity and specificity of at least 1.5 [41].

Statistical analysis was performed using R [42] software and Microsoft Excel 2016 (Microsoft Corp, USA). Researchers interested in access to the code used for this research may contact the corresponding author via email. It can take some months to negotiate access to the code. The author will assist with any reasonable replication attempts for 2 years following publication.

### 2.4. Ethical Considerations

All HVNs, raters, and RNs involved in the study were informed about the purpose of the study, methods, safety considerations, and their right to withdraw consent at any time; they provided consent for participation. This study was approved by the Research Ethics Committee of the Graduate School of Medicine, the University of Tokyo (approval number: 2021319NI-(3)).

## 3. Results

### 3.1. Development of the Checklists

#### 3.1.1. FGIs

Two mentors and four novice HVNs participated. Through this process, four requirements were extracted from the mentors in order to allow HVNs providing nursing care during their visits. The requirements were as follows: (1) the nurses concerned can say that they have experience in performing and can perform the target skill, (2) the nurse concerned is able to safely perform the target skill, (3) the nurse concerned is able to judge the onset of abnormalities, and (4) the nurse concerned can be contacted for each instance when an abnormality or disturbance occurs. Therefore, checklists and each item on them were revised and re-formatted in accordance with these requirements, and each item was classified according to which of the four requirements it was intended to assess.

In addition, feedback from the FGIs indicated that achieving “satisfactory” on all items was too hard and not aligned with the current criteria of “independent” in a clinical environment. Nevertheless, all items were recognized as important for providing high-quality nursing care and should be achieved at a later stage. In response to this, we also categorized the checklist items into two groups: “required items,” in which HVNs must be able to provide appropriately to conduct safe care, and “recommended items,” which are not essential but still important for providing better nursing care. Each item in the drafts was then categorized as either “required items” or “recommended items” following the opinions obtained from FGIs.

Consequently, refined checklists consisted of 19, 22, 25, and 22 items for PIVC placement, pressure injury care, defecation care, and skin tear care, respectively. Among these, the numbers of required items were 9, 16, 6, and 5, respectively.

#### 3.1.2. NGT

The group-meeting participants comprised four administrators from HVN agencies and three nursing researchers. Two administrators were either certified nurses in visiting nursing or wound, ostomy, and continence nursing, and the researchers included a professor with expertise in geriatric nursing and wound management, an associate professor who is a certified nurse in home care with expertise in home care nursing, and an assistant professor with expertise in home care nursing.

After we reached consensus, the developed checklists consisted of 19 items for PIVC placement and pressure injury care, and 21 items for defecation care and skin tear care. Supporting 1 shows the final version of the developed checklists for each nursing skill. The developed checklists were used to assess the HVNs' nursing skills during their visits through the video recordings of care provided. The expected raters of the checklists were nurses who were competent enough to play the role of mentor nurses. Each item was divided into steps, and the item description explained how each step should be performed to provide appropriate care, which should be assessed as “satisfactory,” “unsatisfactory,” “inapplicable,” or “unassessable.” HVNs who were assessed “required items” as “satisfactory” were considered as HVNs who were “independent,” which means that the HVNs were skilled enough to provide nursing care during their visits. In the cases where there were items assessed as “inapplicable” for the required items, when providing treatment that corresponds to such items, it is preferable to perform a skill assessment using the checklist again at the subsequent rounds.

#### 3.1.3. Participants' Characteristics

Six nursing researchers enrolled as raters. One of the raters lost contact during the evaluations; therefore, we excluded the rater from the analysis and five researchers' evaluations were included in the study.

The characteristics of the raters are described in Table 1. The median and interquartile range of clinical experience was 5.0 (4.5–12.0) years, worked as a mentor for RNs for 1.0 (0.0–3.0) years, and had 6.5 (6.0–8.0) years of experience as an educator for nursing students. The participants who had a certain expertise in nursing care, particularly had sufficient proficiency to conduct the care independently and had at least one publication related to the care, played the role of a reference rater for each care.

#### 3.1.4. Results of the VBA Using the Checklists

Six videos of PIVC placement and skin tear care, and seven videos of pressure injury and defecation care were assessed by the raters. The proportion of videos assessed as “independent” by more than three raters were 33.3%, 14.3%, 57.1%, and 66.7% for PIVC placement, pressure injury care, defecation care, and skin tear care, respectively.

#### 3.1.5. Reliability and Concurrent Validity of the Overall Results

The inter- and intrarater reliabilities, sensitivity, and specificity of the overall assessments for pressure injury care, skin tear care, defecation care, and PIVC placement based on the checklists are shown in Table 2. The AC1 values, indicating both the overall inter- and intrarater reliabilities of the checklists, ranged from 0.72 to 1.00. The sensitivity and specificity ranged from 0.90 to 1.00 and 0.93 to 1.00, respectively.

#### 3.1.6. Reliability and Concurrent Validity of Each Item of the Checklist

The inter- and intrarater reliability of each item on the checklist is shown in Table 3. The AC1 values, indicating the items' inter-rater reliability of PIVC placement, pressure injury care, defecation care, and skin tear care, ranged from 0.63 to 1.00, 0.68 to 1.00, 0.72 to 1.00, and 0.67 to 1.00, respectively. The values of AC1, indicating the items' intrarater reliability of PIVC placement, pressure injury care, defecation care, and skin tear care, ranged from 0.76 to 1.00, 0.63 to 1.00, 0.76 to 1.00, and 0.76 to 1.00, respectively. In addition, the number of items with 100.0% of “satisfactory” was seven, one, five, and seven for PIVC placement, pressure injury care, defecation care, and skin tear care, respectively.

## 4. Discussion

This study developed objective VBA checklists for HVNs' nursing skills, and the results showed that the developed checklists possessed high concurrent validity and inter-/intrarater reliabilities, with more than substantial inter- and intrarater reliabilities for each item of the checklist, according to Landis and Koch's benchmark [40]. Since VBA does not require accompanying visits and videos can be recorded during the nurses' visits without disturbing the care provided, the checklists may provide an easy, useful, and less human resource and cost-effective assessment of nursing skills for HVNs. This would further help to conduct quality assurance in HVN agencies.

Through the literature review, FGI, and group meeting using NGT, we have developed checklists that cover the entire procedure of nursing skills; however, given the number of items in the checklists, the evaluation process can be time-consuming. To address this, future studies might be worth considering employing a Global Rating Scale or a shortened version of the developed checklists (e.g., scoring only the “required items”), as the Global Rating Scale is also a reliable and easily implementable method that may allow for more efficient assessment [43]. In addition, recent studies have employed video analysis using deep learning to automate the assessment of surgical procedures [23, 24], and their findings suggest the potential for accurate, objective evaluations. Although differences in clinical settings pose challenges to the immediate application of automated VBA, such automation could improve the efficiency of assessment and feedback delivery. Therefore, the development of automated VBA should also be considered as a promising future direction to minimize mentors' workload.

While high reliability was confirmed for most items in the developed checklists, relatively low reliability was observed for a few items (Table 3). This could be because the descriptions of those items included abstract explanations, such as “an abundant amount of water is used in accordance with the pocket size” in pressure injury item no. 13 (Supporting 1). Since the absence of operational definitions leads to measurement error [44], these items might have been rater-dependent. We avoided abstract descriptions; however, several items were unavoidable due to the need for patient condition–dependent judgments. In addition, although previous studies have suggested that a first-person perspective, which can capture more detailed procedural steps than third-person perspectives, subtle movements conducted with a glance or outside the recording range may have been missed by the raters, potentially lowering inter-rater reliability [45].

Furthermore, the specificity was higher than the sensitivity in the developed checklists. The results suggest that the reference raters' subjective assessments tended to be less concrete than the checklist assessments. Through the NGT and FGIs, we extracted “ideal requirements”; however, clinical decisions were thought to be made through comprehensive assessments. This resulted in “satisfactory” or “unsatisfactory” ratings, according to each item being assessed more strictly.

Despite the recordings contributing to the improvement of the safety profile and proficiency level [46, 47], video recording during medical procedures involves ethical issues with respect to privacy [48], such as filming patients and their living environments. In addition, there is a threat of losing data during the visits. To address these concerns, it is essential to pay careful attention to minimizing the risk of violating patients' privacy and dignity while maximizing the benefits by establishing clear regulations within legal and institutional frameworks. In the meantime, HVNs should rigorously assess the necessity of recording and take all possible precautions, such as obtaining informed consent from patients and/or guardians, ensuring secure data management, and restricting access strictly to mentors. Further research and discussions are required to examine the benefits and safe implementation of the VBA, which may facilitate future advancements in policy and technology to support its broader implementation.

This study had some limitations. First, since the videos used for the assessments were taken with simulated patients, this study design may have affected the generalizability of our findings. Second, although the reference raters were nominated based on their knowledge, skills, experience, and achievements related to nursing skills, the reference assessment could be biased by the reference raters. Third, some raters were involved in the study, which may have contributed to potential bias. Fourth, the developed checklists assume that the mentor has a certain level of competency; thus, it is unclear whether nurses who have a limited level of competence can be appropriately assessed through videos and checklists. Fifth, even though we ensured the content validity of the checklists, whether HVNs assessed as skilled enough can provide safe care remains unclear. Considering these limitations, further large-scale studies in diverse clinical settings using blinded external raters with hard patient outcomes, such as the occurrence of adverse events, are required to enhance the generalizability of the results.

## 5. Conclusions

This study showed high inter- and intrarater reliabilities and concurrent validity of the developed checklists. VBA is a promising method for assessing nursing skills, especially for HVNs, as a limited number of opportunities exist to receive nursing skill assessments. These findings suggest the possibility of implementing VBA in home care and contribute to improving the quality of care by enabling burdenless quality assurance for nursing skills.

## Figures and Tables

**Table 1:** Characteristics of raters.

	Rater (*N* = 5)
Age (years)	37.0 [36.0–38.0]
Gender (female)	3 (60.0)
Clinical experience (years)	5.0 [4.5–12.0]
Experience as a supervisor for RNs (years)	1.0 [0.0–3.0]
Experience as an educator for nursing students (years)	6.5 [6.0–8.0]

*Note:* Data are presented as *n* (%) or median [interquartile range].

Abbreviation: RN, registered nurse.

**Table 2:** Overall reliability and concurrent validity of developed checklists.

		Inter-rater reliability (*N* = 5)	Intrarater reliability (*N* = 5)	Sensitivity (*N* = 5)	Specificity (*N* = 5)
PIVC placement	(*n* = 6)	0.76	0.82	0.90	0.95
Pressure injury care	(*n* = 7)	1.00	1.00	1.00	1.00
Defecation care	(*n* = 7)	0.72	0.72	0.90	0.93
Skin tear care	(*n* = 6)	0.81	0.81	0.90	1.00

*Note: N* = number of raters; *n* = number of videos used for the statistical analysis. PIVC: peripheral intravenous catheter.

**Table 3:** Checklist items' inter-rater reliability and intrarater reliability.

**PIVC placement**					**Pressure injury care**					**Defecation care**					**Skin tear care**				
**Items**		**Inter-rater reliability**	**Intrarater reliability**	**Proportion of satisfactory**	**Items**		**Inter-rater reliability**	**Intrarater reliability**	**Proportion of satisfactory**	**Items**		**Inter-rater reliability**	**Intrarater reliability**	**Proportion of satisfactory**	**Items**		**Inter-rater reliability**	**Intrarater reliability**	**Proportion of satisfactory**
		**(*n* = 30)**	**(*n* = 60)**	**(*n* = 30)**			**(*n* = 35)**	**(*n* = 70)**	**(*n* = 35)**			**(*n* = 35)**	**(*n* = 70)**	**(*n* = 35)**			**(*n* = 30)**	**(*n* = 60)**	**(*n* = 30)**
1.	Practical experience and self-assessment	1.00	1.00	100.00	1.	Practical experience and self-assessment	1.00	1.00	100.00	1.	Practical experience and self-assessment	1.00	1.00	100.00	1.	Practical experience and self-assessment	1.00	1.00	100.00
2.	Assessment prior to drip infusion administration	0.89	0.92	93.33	2.	Risk assessment for pressure injuries	0.91	0.79	25.71	2.	Medical history taking	0.90	0.97	94.29	2.	Observation of common sites of skin tears	0.76	0.96	90.00
3.	Confirmation of the order for the infusion	0.74	0.76	86.67	3.	Assessment of depth using the DESIGN-R 2020 tool	1.00	1.00	85.71	3.	Visual examination	1.00	0.87	100.00	3.	Assessment of dry skin	1.00	0.93	100.00
4.	Preparation of the required materials	1.00	1.00	100.00	4.	Assessment of exudate using the DESIGN-R 2020 tool	1.00	1.00	85.71	4.	Auscultation	0.93	0.93	88.57	4.	Assessment of skin thinning	0.90	0.86	80.00
5.	Hand hygiene	0.89	0.92	93.33	5.	Assessment of size using the DESIGN-R 2020 tool	1.00	1.00	85.71	5.	Percussion test/ultrasonography	0.90	0.94	91.43	5.	Assessment of blisters and blood blisters	1.00	0.91	83.33
6.	Infusion preparation: mixing	1.00	1.00	100.00	6.	Assessment of inflammation/infection using the DESIGN-R 2020 tool	1.00	0.95	57.14	6.	Palpation	1.00	0.94	100.00	6.	Assessment of senile purpura	1.00	0.97	100.00
7.	Infusion preparation: priming	0.80	0.83	90.00	7.	Assessment of granulation tissue using the DESIGN-R 2020 tool	1.00	0.92	85.71	7.	Palpation/rectal examination/ultrasonography	0.88	0.97	94.29	7.	Assessment of pseudoscar	1.00	0.85	100.00
8.	Patient identification	1.00	0.93	100.00	8.	Assessment of necrotic tissue using the DESIGN-R 2020 tool	1.00	1.00	85.71	8.	Physical examination order	1.00	1.00	100.00	8.	Assessment of edema	0.78	0.78	73.33
9.	Explanation of the subject	1.00	0.89	100.00	9.	Assessment of pocket using the DESIGN-R 2020 tool	1.00	1.00	85.71	9.	Assessment of emergencies	0.94	0.91	97.14	9.	Assessment of contracture	0.67	0.76	73.33
10.	Selection of the paracentesis site	1.00	1.00	100.00	10.	Assessment of deterioration	1.00	0.96	85.71	10.	Assessment of constipation	0.94	0.97	97.14	10.	Implementation of care for skin tear prevention	0.91	0.96	86.67
11.	Selection of the blood vessel using ultrasound	1.00	0.94	33.33	11.	Careful removal of tape (including dressings with an adhesive part)	0.77	0.86	25.71	11.	Assessment of the cause of constipation	0.80	0.91	91.43	11.	Education on care for skin tear prevention	1.00	1.00	100.00
12.	Execution of ultrasound-guided puncture	1.00	0.94	33.33	12.	Cleansing of the wound and peri-wound surface	0.74	0.72	22.86	12.	Assessment of the ability to pass stools	0.83	0.91	91.43	12.	Judgment and treatment at skin tear onset	0.93	0.89	96.67
13.	Ultrasound execution after placement	0.91	0.91	30.00	13.	Cleansing of the pocket	0.68	0.95	37.14	13.	Selection of appropriate care for defecation	0.83	0.97	91.43	13.	Assessment of the skin flap color	0.89	0.92	93.33
14.	Execution of puncture	1.00	0.93	100.00	14.	Water removal after cleansing	0.71	0.85	71.43	14.	Defecation guidance	1.00	1.00	100.00	14.	Assessment of the ability of skin flap realignment	0.91	0.96	86.67
15.	Indwelling catheter fixation	0.82	0.92	36.67	15.	Nonuse of a disinfectant	1.00	0.96	85.71	15.	Guidance about defecation posture	0.93	1.00	11.43	15.	Collection of information regarding the onset background	0.93	0.97	96.67
16.	Adjustment of the number of drops	0.71	0.91	80.00	16.	Application of a dressing	0.87	0.96	77.14	16.	Guidance to promote defecation	0.89	0.94	54.29	16.	Assessment of skin tear pain	0.85	0.80	93.33
17.	Instructions at the time of drip infusion administration	0.68	0.77	80.00	17.	Application of a tape (including dressing with an adhesive part)	0.81	0.63	28.57	17.	Abdominal massage	0.72	0.76	74.29	17.	Compression hemostasis	1.00	1.00	100.00
18.	Observation of adverse events	0.87	0.89	86.67	18.	Treatment of infected pressure injuries	0.91	1.00	74.29	18.	Implementation of fecal disimpaction	0.79	0.83	80.00	18.	Careful removal of tape (including dressings with an adhesive part)	0.78	0.89	6.67
19.	Management of medical waste	0.63	0.85	83.33	19.	Judgment at the onset of an abnormality	1.00	1.00	85.71	19.	Implementation of glycerin enema	1.00	1.00	0.00	19.	Wound irrigation	1.00	1.00	100.00
										20.	Execution of suppository insertion	1.00	0.87	0.00	20.	Skin flap care	0.93	1.00	96.67
										21.	Changing diapers	0.76	0.84	88.57	21.	Wound protection	0.78	0.83	73.33

*Note: n* = number of evaluations used for the statistical analysis. PIVC: peripheral intravenous catheter.

## Data Availability

The data that support the findings of this study are available from the corresponding author upon reasonable request.

## Supporting Information

Supporting file: the video-based assessment checklists of peripheral intravenous catheter placement, pressure injury care, defecation care, and skin tear care for home-visiting nurses.
