# Supplementary material for: Development and Evaluation of the Reliability and Validity of Video-Based Assessment Checklists of Nursing Skills via Chest-Mounted Cameras for Home-Visiting Nurses
Source: Nurs Res Pract. 2025 Oct 23;2025:7893018. doi: 10.1155/nrp/7893018 (PMC12575045; doi:10.1155/nrp/7893018)
Supplement: Supporting Information — Additional supporting information can be found online in the Supporting Information section. [file 7893018.f1.docx]

**Video-based assessment checklist for home-visiting nurses to become independent
(Skill:** **Peripheral intervenous catheter placement)**

Main indicators

The assessment and successful experience in paracentesis to date

Basic standards for becoming independent (Concepts)

1. The nurse concerned can say that they have experience in performing and can perform the target skill.

2. The nurse concerned is able to perform the target skill safely.

3. The nurse concerned is able to judge the onset of abnormalities.

4. The nurse concerned can be contacted for each instance when an abnormality or disturbance occurs.

| Required | Classification | Checklist item | Item explanation | Assessment method | Correspondence to concept |
| --- | --- | --- | --- | --- | --- |
| ★ | Self-assessment | Practical experience and self-assessment | The individual concerned has practical experience and believes that he/she is competent. | Record | 1 |
|  | Pre-confirmation and preparation | Assessment prior to drip infusion administration | Before administering a infusion, vital sign measurement and medical interview are performed. If either is carried out, the item will be considered satisfactory. | Video | 3,4 |
|  |  | Confirmation of the order for the infusion | The patient’s name, drug name, administration method, and administration duration are confirmed by pointing and calling out. | Video | 2 |
| ★ |  | Preparation of the required materials | All required materials are prepared by the start of the infusion procedure. | Video | 2 |
| ★ |  | Hand hygiene | If all of the following conditions are met by the relevant timing, the item will be considered satisfactory:   1. Hand hygiene is maintained by washing hands with running water and soap that lathers or a quick-drying hand rub such as alcohol. 2. Disposable gloves for medical use are used. | Video | 2 |
|  |  | Infusion preparation: mixing | When the infusion needs to be mixed, the rubber stopper is disinfected, after which the total amount of the prescribed drug is dissolved, and the required amount is mixed into the infusion. When the infusion does not need to be mixed, the item will be considered satisfactory. | Video | 2 |
|  |  | Infusion preparation: priming | If all of the following conditions are met, the item will be considered satisfactory. When an infusion set is not used and priming is not necessary, the item will be considered satisfactory.   1. The rubber stopper is disinfected. 2. The appropriate infusion set is selected. 3. The infusion chamber is filled with an adequate amount of the drug solution. 4. The drug solution is filled to the tube tip so that no air can enter. | Video | 2 |
|  |  | Patient identification | Before administration, the patient’s full name and date of birth is confirmed with the patient concerned and/or their caregiver, and patient identification is performed by pointing and calling out. | Video | 2 |
| ★ |  | Explanation to the subject | Before administration, the infusion purpose, contents, precautions during administration, and actions are explained verbally to the patient concerned and/or their caregiver. | Video | 2 |
| ★ | Selection | Selection of the paracentesis site | Sites with a high risk of nerve damage, such as those within 5 cm of the styloid process of radius with superficial branches of the radial nerve, and contraindicated sites, such as the arm on the shunt side, are not selected. | Video | 2 |
|  |  | Selection of the blood vessel using ultrasound | Ultrasound is performed upon selecting a blood vessel with appropriate thickness and depth. When ultrasound is not used, the item will be considered inapplicable. | Video | 2 |
|  | Paracentesis | Execution of ultrasound-guided puncture | The catheter is punctured while observing the blood vessel via ultrasound. When ultrasound is not used, the item will be considered inapplicable. | Video | 2 |
|  |  | Ultrasound execution after placement | The puncture site is observed by ultrasound after placement. When ultrasound is not used, the item will be considered inapplicable. | Video | 2 |
| ★ |  | Execution of puncture | The puncture is attempted only two or fewer times, and backflow is confirmed. Even when it is unverifiable on the video whether the novice nurse checked backflow, when it is verifiable by the novice nurse’s words, the item will be considered satisfactory. When paracentesis fails two or more times, the item will be considered unsatisfactory. | Video | 2 |
| ★ | Fixation | Indwelling catheter fixation | If all of the following conditions are met, the item will be considered satisfactory. Even if they are not performed, the item will be considered satisfactory if the assessor judges that it was clinically difficult to perform.   1. The indwelling needle is fixated using film.   However, when one or more of the following items are confirmed, then the item will be considered unsatisfactory.   1. At the start of fixation, the tourniquet was still in use. 2. Before completing fixation, excessive force was applied that caused the base of the catheter to move greatly. 3. After completing fixation, a loose loop was not formed to prevent kinking of the route. 4. After completing fixation, two places or more, including the puncture site, were not fixated. 5. After completing fixation, the catheter connector was next to the skin.   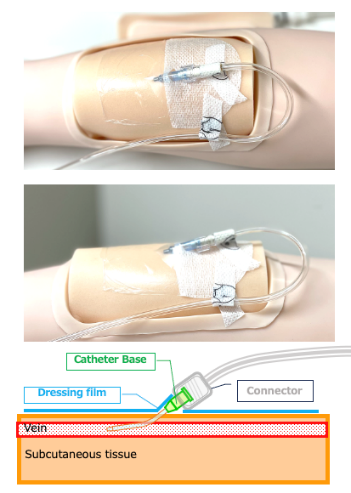 | Video | 2 |
| ★ | Administration | Adjustment of the number of drops | When all of the following conditions are met to perform the adjustments, then the item will be considered satisfactory. Confirmation of the drip speed is not performed here.   1. The number of drops is adjusted according to the drip volume and time instructed by the physician.   When making adjustments, the indwelling catheter site is not elevated and the joints nearby are not bent. | Video | 2 |
|  |  | Instructions at the time of drip infusion administration | The patient is informed when insertion and fixation are completed, and is given instructions about precautions to take during the infusion. | Video | 2 |
|  |  | Observation of adverse events | If any of the following are performed after the start of administration, the item will be considered satisfactory:   1. The presence or absence of pain is verified. 2. The indwelling catheter site is monitored.   Adverse events are monitored. | Video | 3,4 |
|  |  | Management of medical waste | If any of the following conditions are met, then the item will be considered satisfactory:   1. Medical waste, such as needles and drug ampoules, are not disposed of as household rubbish. 2. A medical waste box is provided. 3. An indwelling needle with needlestick injury safeguard mechanism is used, and the mechanism is activated. 4. Medical waste is placed in one bag or container.   However, when one of the following is confirmed, the item will be considered unsatisfactory.   1. Medical waste is disposed of together with general waste. 2. An indwelling needle is recapped.   Only when all of the above have not been observed, the item will be considered “undeterminable.” | Video | 2 |
| The assessor will assess each checklist item as “satisfactory” or “unsatisfactory,” and when all required items are assessed to be “satisfactory,” the nurse will have passed the assessment (and become independent). In such instances, “inapplicable” items will be excluded.  When the assessment is “passed” in terms of care including “inapplicable items,” when providing treatment that corresponds to such items, it is preferable to perform a skill assessment using the checklist again. | | | | | |

**Video-based assessment checklist for home-visiting nurses to become independent (Skill: Pressure injury care)**

Main indicators

The assessment and required care based on the assessment can be performed with the same level of skill as the assessor

Basic standards for becoming independent (Concepts)

1. The nurse concerned can say that they have experience in performing and can perform the target skill.

2. The nurse concerned is able to perform the target skill safely.

3. The nurse concerned is able to judge the onset of abnormalities.

4. The nurse concerned can be contacted for each instance when an abnormality or disturbance occurs.

| Required | Classification | Checklist item | Item explanation | Assessment method | Correspondence to concept |
| --- | --- | --- | --- | --- | --- |
| ★ | Self-assessment | Practical experience and self-assessment | The individual concerned has practical experience and believes that he/she is competent. | Record | 1 |
|  | Assessment of risk factors | Risk assessment for pressure injuries | The assessment of risk factors is consistent with that of the assessor. | Record | 3 |
|  | Assessment of pressure injury sites | Assessment of depth using the DESIGN-R^®^ 2020 tool | The assessment for either d, D, DDTI, or DU is consistent with that of the assessor. | Video + record | 3 |
| ★ |  | Assessment of exudate using the DESIGN-R^®^ 2020 tool | The E-score is consistent with that of the assessor. | Video + record | 3 |
|  |  | Assessment of size using the DESIGN-R^®^ 2020 tool | The long-axis and short-axis of pressure injuries are measured, and the S-score is consistent with that of the assessor. | Video + record | 3 |
| ★ |  | Assessment of inflammation/infection using the DESIGN-R^®^ 2020 tool | The I-score is consistent with that of the assessor. | Video + record | 3 |
|  |  | Assessment of granulation tissue using the DESIGN-R^®^ 2020 tool | The assessment for g/G is consistent with that of the assessor. | Video + record | 3 |
| ★ |  | Assessment of necrotic tissue using the DESIGN-R^®^ 2020 tool | The N-score is consistent with that of the assessor. | Video + record | 3 |
|  |  | Assessment of pocket using the DESIGN-R^®^ 2020 tool | The long-axis and short-axis of the pocket is measured, and the P-score is consistent with that of the assessor. | Video + record | 3 |
| ★ |  | Assessment of deterioration | The assessment of deterioration (or no deterioration) in the DESIGN-R^®^ 2020 items is consistent with that of the assessor. | Video + record | 4 |
|  | Wound care | Careful removal of tape (including dressings with an adhesive part) | Tape (including dressings with an adhesive part) is removed using any of the following methods. However, even if another method is used depending on the starting point of tape removal and the type of tape, the item will be considered satisfactory when the assessor judges that “skin damage by tape removal has been prevented.”   1. Pressing on the skin with the hand, the tape is peeled off in a backward direction at a 180° angle. 2. When a film dressing is used, the film dressing is peeled off by pressing the skin with the hand, and lifting and stretching out the dressing in a manner horizontally to the skin. 3. Removal is performed upon reducing adhesiveness such as by applying a release agent between the tape and the skin.   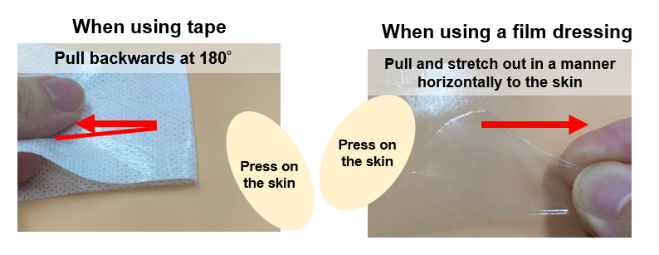 | Video | 2 |
| ★ |  | Cleansing of the wound and peri-wound surface | If both of the following conditions are met, the item will be considered satisfactory.  Instances of pressure injuries with intact skin will be considered inapplicable.   1. The wound area and the skin extending approximately 5–10 cm outwards from the wound edge are washed with soap lather. 2. The wound and its surroundings (area washed using soap) are rinsed with abundant water (tap water or normal saline) in accordance with the wound size and condition.   When the following apply, the item will be considered unsatisfactory.   1. An insufficient amount of water is used for cleansing of a pressure injury with suspected infection or critical colonization. | Video | 2 |
| ★ |  | Cleansing of the pocket | If all of the following conditions are met, the item will be considered satisfactory. If there is no pocket, the item will be considered inapplicable.   1. A catheter or long, thin nozzle is inserted to the tip of the pocket and cleansing is performed. In environments without a catheter, etc., rinsing is performed with strong pressure applied manually to the irrigation bottle to wash the pocket up to the tip. 2. The direction of the catheter and nozzle is adjusted to correspond with the pocket direction, and cleansing is performed using method 1. 3. An abundant amount of water (tap water or normal saline) is used in accordance with the pocket size.   However, even if the conditions above are met, if any of the following apply, the item will be considered unsatisfactory.   1. In part of or throughout the entire pocket there are areas that are not washed to meet the conditions above. 2. An insufficient amount of water is used for cleansing of a pressure injury with suspected infection or critical colonization. | Video | 2 |
| ★ |  | Water removal after clenasing | If both of the following conditions are met, the item will be considered satisfactory:   1. When removing water after cleansing using gauze, area without necrotic tissue are wiped without rubbing. 2. If water remains in the pocket, efforts are made to prevent water from remaining in the pocket, such as by repositioning the body to face the opposite side (if in the left lateral position, repositioning the patient to the right lateral position). | Video | 2 |
| ★ |  | Nonuse of a disinfectant | Except when instructed, a disinfectant is not used for pressure injuries. | Video | 2 |
| ★ |  | Application of a dressing | At sites where pressure is applied to wounds, a thin dressing is applied. For pressure injuries at sites where pressure cannot be applied, such as the thoracic and knees in patients who cannot adopt the prone position, the item is considered inapplicable. | Video | 2 |
| ★ |  | Application of tape (including dressing with an adhesive part) | If all of the following conditions are met, the item will be considered satisfactory:   1. When applying tape, it is done so in a manner so as not to pull the skin. 2. In the event of wrinkles and sagging of the skin, the tape is applied while pulling the wrinkles and lifting the skin. 3. The tape is applied so as not to leave a gap between the skin and the gauze. 4. In the sacral–coccygeal area, tape is applied to prevent lifting at the intergluteal cleft to prevent watery stools from getting in. 5. At sites where external pressure is applied, tape is applied to the four sides of the gauze so that it does not slip.   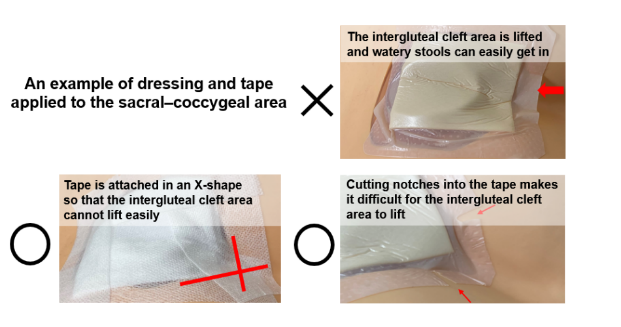 | Video | 2 |
| ★ |  | Treatment of infected pressure injuries | If infection is present, a sealed dressing, such as with a film dressing, is not used, except when using a silver-containing dressing for infection control. | Video | 2 |
| ★ | Report at the onset of an abnormality | Judgment and report at the onset of an abnormality | When there is wound deterioration, the judgment to change the care and treatment method and consultation with other staff members is made an appropriate manner.  In cases where, upon viewing the video, the assessor judges it necessary to change the treatment and to consult and report the case, if “no change is required” or “reporting is unnecessary” is recorded, then the item will be considered unsatisfactory. | Record | 4 |
| The assessor will assess each checklist item as “satisfactory” or “unsatisfactory,” and when all required items are assessed to be “satisfactory,” the nurse will have passed the assessment (and become independent). In such instances, “inapplicable” items will be excluded.  When the assessment is “passed” in terms of care including “inapplicable items,” when providing treatment that corresponds to such items, it is preferable to perform a skill assessment using the checklist again. | | | | | |

**Video-based assessment checklist for home-visiting nurses to become independent (Skill: Defecation care)**

Main indicators

The assessment and required care based on the assessment can be performed with the same level of skill as the assessor

Basic standards for becoming independent (Concepts)

1. The nurse concerned can say that they have experience in performing and can perform the target skill.

2. The nurse concerned is able to perform the target skill safely.

3. The nurse concerned is able to judge the onset of abnormalities.

4. The nurse concerned can be contacted for each instance when an abnormality or disturbance occurs.

| Required | Classification | Checklist item | Item explanation | Assessment method | Correspondence to concept |
| --- | --- | --- | --- | --- | --- |
| ★ | Self-assessment | Practical experience and self-assessment | The individual concerned has practical experience and believes that he/she is competent. | Record | 1 |
| ★ | Assessment of the abdominal region | Medical history taking | The necessary information pertaining to defecation (subjective symptoms, status of oral medication-taking, and living conditions) is successfully collected. If the assessor judges that the necessary information is not collected, then the item will be considered unsatisfactory. | Video + record | 2 |
|  |  | Visual examination | Abdominal distension, mass, distention, hemorrhoids, anal fissures, anal swelling, rectal prolapse, and stool properties (Bristol stool form scale) are assessed by visual examination. | Video + record | 2 |
|  |  | Auscultation | The presence or absence of bowel sounds and metallic sounds is assessed by auscultation. | Video + record | 2 |
|  |  | Percussion test/ultrasonography | The presence or absence of gas and gas distribution is assessed by percussion test/ultrasonography. | Video + record | 2 |
|  |  | Palpation | The presence or absence of tenderness, rebound tenderness, and muscular defense is assessed by palpation. | Video + record | 2 |
|  |  | Palpation/rectal examination/ ultrasonography | The presence or absence of fecal masses and distribution thereof is assessed by palpation/rectal examination/ultrasonography. | Video + record | 2 |
|  |  | Physical examination order | Any physical examination (history taking, visual examination, auscultation, and percussion test) is performed prior to deep palpation. When deep palpation is performed without performing a single physical examination, the item will be considered unsatisfactory, in other cases, the item will be considered satisfactory. | Video + record | 2 |
| ★ | Assessment of emergency symptoms | Assessment of emergencies | The presence or absence of emergency items that should be reported to the physician, such as acute abdomen, intestinal obstruction, ileus, and hemorrhage of the digestive tract, are verified based on the physical examination, and reporting is performed when deemed necessary. In cases that do not require consultation or reporting, the item will be considered satisfactory. In cases where the assessor judges it necessary to perform a change in treatment, and consultation or reporting based on the video but “change is not needed” or “reporting unnecessary” is recorded, the item will be considered unsatisfactory. | Video + record | 3,4 |
| ★ | Assessment of suspected constipation | Assessment of constipation | Suspected constipation is assessed based on the physical examination and bowel movement record. | Video + record | 2,3 |
|  | Classification of constipation | Assessment of the cause of constipation | When constipation is suspected, an assessment is performed to determine whether dyschezia (defecation disorder), decreased defecation frequency (transit disorder), or normal (no constipation) applies. | Video + record | 2 |
|  | Assessment of the ability to pass stools | Assessment of the ability to pass stools | The ability to pass stools is assessed according to the following that applies:   1. Stools can be passed by oneself regardless of incontinence and the state of drug usage. 2. Stools can be passed by oneself with support, such as toileting assistance and abdominal massage. 3. Stools can be passed with the use of either fecal disimpaction, enema, or suppositories. | Record | 2 |
| ★ | Selection of necessary care | Selection of appropriate care for defecation | Care for defecation is selected according to an assessment, and the selection is consistent with that of the assessor. | Video + record | 2 |
|  | Defecation care | Defecation guidance | When ambulation is possible, if the following conditions are met, then the item will be considered satisfactory.   1. Assistance is provided to move the patient to the toilet while taking due care of falling. 2. A portable toilet is prepared as needed.   However, when any of the following apply, the item will be considered satisfactory regardless of the presence or absence of having carried out the item.   1. Implementation is offered. 2. Defecation has already been attempted, and there is no need for additional defecation guidance. 3. Patient ambulation is difficult. | Video | 2 |
| ★ |  | Guidance about defecation posture | When the patient can sit on a toilet, support and guidance is provided, such as to stabilize the feet to be able to adopt the correct defecation posture, to lean forward easily, and to easily apply abdominal pressure. When guidance for defecation posture is not performed, the item will be considered inapplicable. | Video | 2 |
|  |  | Guidance to promote defecation | Regarding selection of the required defecation care, the following three items are assessed: “guidance for meal content, and the volume of water intake,” “guidance for exercise and sleep,” and “the importance of laxative control.” Furthermore, an assessment will be considered to have been performed only when at least one of these three items is recorded, and by doing so, the item will be considered satisfactory. If there is no record of the aforementioned three items, and the assessor judges that there is no need for the abovementioned care, then the item will be considered satisfactory. | Video + record | 2 |
| ★ |  | Abdominal massage | If the following conditions are met, then the item will be considered satisfactory.   1. Abdominal massage is performed.   When the condition above is not verified, the item will be considered satisfactory if any of the following are performed.   1. Pressure is applied on the lower abdomen during fecal disimpaction. 2. Deep palpation is performed. 3. The novice nurse offers to perform an abdominal massage. | Video | 2 |
| ★ |  | Implementation of fecal disimpaction | Paying attention to rectal perforation, fecal disimpaction is performed according to the following procedure while observing the patient’s facial color, abdominal pain, nausea, bleeding, and sudden drop in blood pressure. When fecal disimpaction is not performed after it has been assessed to be necessary, or when a suppository is inserted, or glycerin enema is performed without performing fecal disimpaction, the item will be considered unsatisfactory. However, when fecal disimpaction is not performed after it has been assessed to be unnecessary, the item will be considered inapplicable.   1. Posture is changed to the left lateral position. 2. A lubricant is applied around the anus and a finger. 3. The finger is inserted 4–5 cm into the anus, and the stool that is retained in the lower rectum and that can be evacuated is removed. 4. Pressure is applied to the lower abdominal area when needed.   Reaction feces are confirmed. | Video | 2 |
| ★ |  | Implementation of glycerin enema | Paying attention to rectal perforation, glycerin enema is performed according to the following procedure while observing the patient’s facial color, abdominal pain, nausea, bleeding, and sudden drop in blood pressure. When glycerin enema is not performed, the item will be considered inapplicable.   1. Posture is changed to the left lateral position. 2. The need for glycerin enema is verified by rectal examination and fecal disimpaction. 3. The enema solution is warmed to approximately 40 °C. 4. The cap is removed, and a stopper of 6–8 cm is prepared. 5. A lubricant is applied, a tube of 5–6 cm is inserted, and the enema fluid is slowly infused. 6. If there is resistance, the position of the tube is adjusted. 7. Following infusion, the lower limbs are extended so that the enema fluid does not get discharged immediately. 8. Reaction feces are confirmed. | Video | 2 |
| ★ |  | Execution of suppository insertion | Paying attention to rectal perforation, a suppository is inserted according to the following procedure while observing the patient’s facial color, abdominal pain, nausea, bleeding, and sudden drop in blood pressure. When a suppository is not inserted, the item will be considered inapplicable.   1. Posture is changed to the left lateral position. 2. The need for suppository insertion is verified by rectal examination and fecal disimpaction. 3. The suppository is inserted to a depth of 4–5 cm from the anus (behind the internal sphincter muscle of anus). 4. Expulsion of the suppository from the anus is prevented for 2–3 min by pressing with a gauze and extending the legs. 5. Reaction feces are confirmed. | Video | 2 |
| ★ |  | Changing diapers | After changing the diaper, when there is no clear soiling of the diapers, nightwear, and bedding, the item will be considered satisfactory. When the briefs are not changed, or changing of the diapers are not recorded on the video, then the item will be considered inapplicable. | Video | 2 |
| The assessor will assess each checklist item as “satisfactory” or “unsatisfactory,” and when all required items are assessed to be “satisfactory,” the nurse will have passed the assessment (and become independent). In such instances, “inapplicable” items will be excluded.  When the assessment is “passed” in terms of care including “inapplicable items,” when providing treatment that corresponds to such items, it is preferable to perform a skill assessment using the checklist again. | | | | | |

**Video-based assessment checklist for home-visiting nurses to become independent (Skill: Skin tear care)**

Main indicators

The assessment and required care based on the assessment can be performed with the same level of skill as the assessor

Basic standards for becoming independent (Concepts)

1. The nurse concerned can say that they have experience in performing and can perform the target skill.

2. The nurse concerned is able to perform the target skill safely.

3. The nurse concerned is able to judge the onset of abnormalities.

4. The nurse concerned can be contacted for each instance when an abnormality or disturbance occurs.

**Prevention of skin tears**

| Required | Classification | Checklist item | Item explanation | Assessment method | Correspondence to concept |
| --- | --- | --- | --- | --- | --- |
| ★ | Self-assessment | Practical experience and self-assessment | The individual concerned has practical experience and believes that he/she is competent. | Record | 1 |
|  | Common sites of skin tear onset | Observation of common sites of skin tears | Observation of the skin condition is performed at appropriate sites (the upper and lower extremities, and attachment sites of fixation tape and monitoring electrodes). | Video | 2 |
| ★ | Assessment of risk factors | Assessment of dry skin | The assessment of presence or absence of dry skin (scales on the upper limbs and rhagades on the lower limbs) is consistent with that of the assessor. | Video + record | 3 |
|  |  | Assessment of skin thinning | The assessment of presence or absence of facial tissue-like skin is consistent with that of the assessor. | Video + record | 3 |
|  |  | Assessment of blisters and blood blisters | The assessment of presence or absence of blisters and blood blisters is consistent with that of the assessor. | Video + record | 3 |
|  |  | Assessment of senile purpura | The assessment of presence or absence of senile purpura (subcutaneous bleeding caused by dermal and vascular fragility) is consistent with that of the assessor. | Video + record | 3 |
|  |  | Assessment of pseudoscar | The assessment of presence or absence of pseudoscar (superficial scar tissue and linear or stellate leukoderma caused by healing of superficial trauma or microscopic injury) is consistent with that of the assessor. | Video + record | 3 |
|  |  | Assessment of edema | The assessment of presence or absence of edema is consistent with that of the assessor. | Video + record | 3 |
|  |  | Assessment of contracture | The assessment of presence or absence of contracture from the shoulders to the fingers is consistent with that of the assessor. | Video + record | 3 |
| - In the event that a skin tear is detected, care for the skin tear is provided at such stage (refer to the table for “skin tear care techniques”) | | | | | |
| ★ | Preventive care | Implementation of care for skin tear prevention | Skin tear preventive care (arm cover, bed-rail cover, and moisturizing) is provided based on the risks. If there are no risks of skin tears, it will be considered as “inapplicable.” | Video | 2 |
| ★ | Guidance about prevention methods to the caregiver | Education on care for skin tear prevention | The need for preventive care of skin tears and basic care methods are communicated verbally to the individual concerned or their caregiver, such as a family member. If there is no risk of skin tears, and when preventive care has already been provided and education is thus unnecessary, it will be considered inapplicable. | Video | 2 |
| ★ | Report at the onset of a skin tear detection | Judgment and report at skin tear onset | When cutaneous findings suggesting skin tear are newly detected, it is reported to other staff before treatment and after completion of the home visitation. | Video | 4 |

**Skin tear care techniques**

| Required | Classification | Checklist item | Item explanation | Assessment method | Correspondence to concept |
| --- | --- | --- | --- | --- | --- |
| ★ | Assessment of skin tear | Assessment of the skin flap color using assessment of the ability of skin flap realignment (STAR) classification | The STAR classification selected according to the skin flap color is consistent with that of the assessor. | Video + record | 2 |
|  |  | Assessment of the ability of skin flap realignment using STAR classification | The ability to realign the skin flap to the normal anatomical position is assessed, and the STAR classification selected according to the skin flap is consistent with that of the assessor. | Video + record | 2 |
| ★ | Onset background | Collection of information regarding the onset background | The circumstances surrounding the onset of a skin tear is verified with the individual concerned or their caregiver, such as a family member. Furthermore, verification of environmental factors that can lead to the onset of skin tear. If the skin tear is not new, the item will be considered “satisfactory” even if information collection is not performed. | Video | 3 |
|  | Assessment of skin tear pain | Assessment of skin tear pain | The presence or absence of pain and when pain occurs is verified with the individual concerned. Reporting to the physician and/or instructor is performed as needed. When reporting is not performed in cases in which treatment is changed, and the assessor judges a consultation and reporting to be necessary as seen via video, the item will be considered “unsatisfactory.” | Video | 3, 4 |
| ★ | Wound care | Compression hemostasis | In the event of bleeding, compression hemostasis is performed in an appropriate manner. When there is no bleeding and compression hemostasis is not performed, the item will be considered “satisfactory.” | Video | 2 |
| ★ |  | Careful removal of tape (including dressings with an adhesive part) | Tape (including dressings with an adhesive part) is peeled off using any of the following methods along the direction of the skin flap. When there is no tape applied, such as in new onset, the item will be considered inapplicable.   1. Pressing on the skin with the hand, the tape is peeled off in a backward direction at a 180° angle. 2. When a film dressing is used, the dressing is peeled off by lifting and stretching out in a manner parallel to the skin. 3. Removal is performed using a release agent. |  |  |
| ★ |  | Wound irrigation | The wound is washed using tepid water (physiological saline if possible), and impurities and hematomas are successfully washed away. When irrigation is not performed because the wound has already been cleaned, it will be considered inapplicable. | Video | 2 |
|  |  | Skin flap care | When caring for skin tears in which there is a skin flap and the skin flap has shifted, the skin flap is replaced using an appropriate method. If there is no skin flap or if the skin flap has not shifted and there is no need to put it back in place, mark it as satisfactory. | Video | 2 |
|  |  | Wound protection | The wound dressing suited to the state of the skin tear is selected. | Video | 2 |
| The assessor will assess each checklist item as “satisfactory” or “unsatisfactory,” and when all required items are assessed to be “satisfactory,” the nurse will have passed the assessment (and become independent). In such instance, “inapplicable” items will be excluded.  When the assessment is “passed” in terms of care including “inapplicable items,” when providing treatment that corresponds to such items, it is preferable to perform a skill assessment using the checklist again. | | | | | |
